# Supplementary material for: Co-Extraction of Flaxseed Protein and Polysaccharide with a High Emulsifying and Foaming Property: Enrichment through the Sequence Extraction Approach
Source: Foods. 2023 Mar 16;12(6):1256. doi: 10.3390/foods12061256 (PMC10048294; doi:10.3390/foods12061256)
Supplement: Supplementary file 1 [file foods-12-01256-s001.zip › foods-2234863-supplementary.pdf]

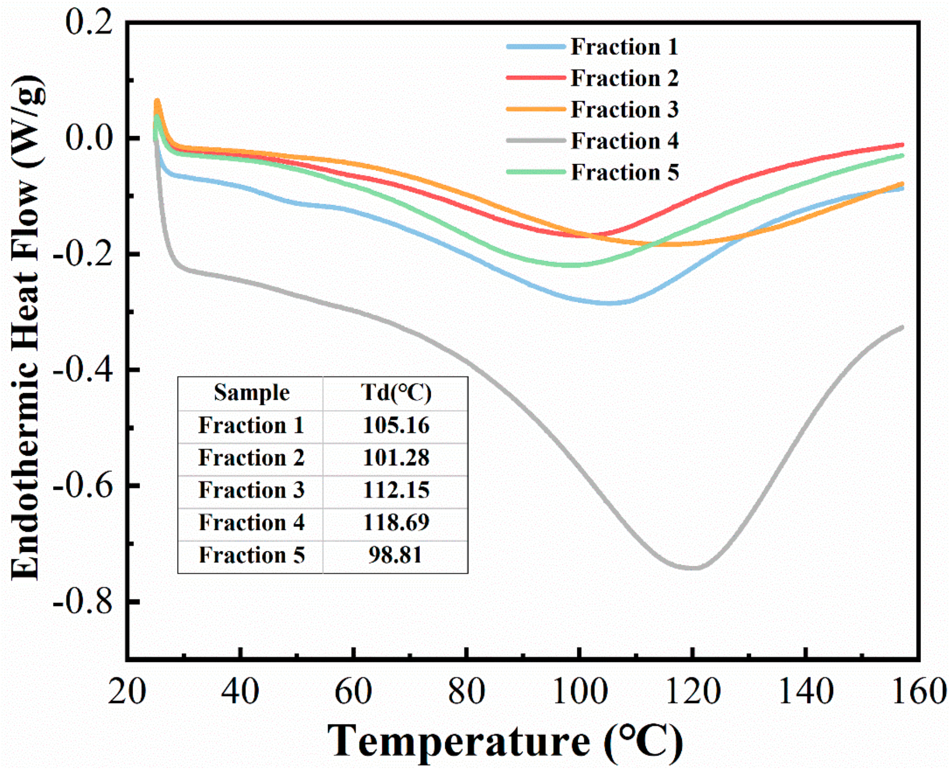

Sample	Td(°C)
Fraction 1	105.16
Fraction 2	101.28
Fraction 3	112.15
Fraction 4	118.69
Fraction 5	98.81

Figure S1. DSC curve and Td (Inserted table) of flaxseed fractions. Td represents denaturation temperature.
